# The complete mitogenome assemblies of 10 diploid potato clones reveal recombination and overlapping variants

**DOI:** 10.1093/dnares/dsab009

**Published:** 2021-07-10

**Authors:** Sai Reddy Achakkagari, Helen H Tai, Charlotte Davidson, Hielke De Jong, Martina V Strömvik

**Affiliations:** 1 Department of Plant Science, McGill University, Montreal, Canada; 2 Fredericton Research and Development Centre, Agriculture and Agri-Food Canada, Fredericton, Canada

**Keywords:** **Key words:** potato mitogenome, mitogenome assembly, recombination

## Abstract

The potato mitogenome is complex and to understand various biological functions and nuclear–cytoplasmic interactions, it is important to characterize its gene content and structure. In this study, the complete mitogenome sequences of nine diploid potato clones along with a diploid *Solanum okadae* clone were characterized. Each mitogenome was assembled and annotated from Pacific Biosciences (PacBio) long reads and 10X genomics short reads. The results show that each mitogenome consists of multiple circular molecules with similar structure and gene organization, though two groups (clones 07506-01, DW84-1457, 08675-21 and H412-1 in one group, and clones W5281-2, 12625-02, 12120-03 and 11379-03 in another group) could be distinguished, and two mitogenomes (clone 10908-06 and OKA15) were not consistent with those or with each other. Significant differences in the repeat structure of the 10 mitogenomes were found, as was recombination events leading to multiple sub-genomic circles. Comparison between individual molecules revealed a translocation of ∼774 bp region located between a short repeat of 40 bp in molecule 3 of each mitogenome, and an insertion of the same in molecule 2 of the 10908-06 mitogenome. Finally, phylogenetic analyses revealed a close relationship between the mitogenomes of these clones and previously published potato mitogenomes.

## 1. Introduction

Plant mitogenomes (mitochondrial genomes) are complex and highly variable in terms of size, structure and organization.^1^ This variability arises mainly from high rates of sequence rearrangements, homologous recombination and repeat content.^1–3^ Despite high variation, mitogenomes are highly conserved in terms of coding sequences as they play a vital role in ATP synthesis and other cellular functions.^4^ Numerous studies have reported the effects of nuclear–cytoplasmic interactions on several traits, especially male sterility.^5–8^ The cytoplasmic male sterility (CMS) is mainly regulated by mitochondrial DNA, and it is important to characterize complete mitogenomes to understand the various effects of nuclear–cytoplasmic interactions.^9^^,^^10^

The plant mitogenome is abundant in repeats that are mainly categorized into three different types; long, short and intermediate-size repeats.^11^ The majority of the recombination events will occur at the large repeat sequences and are reversible, while recombination at short and intermediate-size repeats are very rare, and non-reversible,^11^ making them difficult to detect. Recombination at large repeats, on the other hand, can be easily detected using long-read sequencing data. Previous studies have shown the importance of long reads in determining these rearrangements.^1^ For example, it has been reported that homologous recombination events in potato derived mainly from large repeats of several thousand bp in length.^3^

The potato mitochondrial genome is also complicated and often reported as a multichromosomal type architecture with both circular and linear DNA molecules.^2^^,^^3^ It was shown to exist in the form of multiple individual molecules, and occasionally with shared sequences between them. Recently, three individual molecules in each of 12 potato accessions from diverse taxa were reported.^2^ Similarly, Varré et al.^3^ reported three autonomous molecules with two circular and one linear DNA for two *Solanumtuberosum* cultivars, and Cho et al.^12^ reported five circular DNA molecules in *S.tuberosum* with shared sequences between three of them. Given the diversity within the potato mitogenome, it is necessary to utilize the latest technologies to better understand its landscape of genetic variation, evolution and structural rearrangements.

In the present study, we have used long-read and short-read data to successfully assemble and analyse recombination in nine diploid potato clones (*S. tuberosum*) and one clone of *Solanumokadae*. The results show that these mitogenomes exist as multiple individual molecules with intra-molecular recombination events leading to multiple isoforms and sub-genomic circles. We have also identified key differences in these mitogenomes through comparative analyses. Finally, we have investigated the relationships between these clones and publicly available potato mitogenomes of diverse taxa through phylogenetic analyses.

## 2. Materials and methods

### 2.1. Plant material

A panel was chosen consisting of nine diploid potato clones (07506-01, 12120-03, DW84-1457, 12625-02, 08675-21, H412-1, W5281-2, 11379-03 and 10908-06) and a wild relative *S. okadae* clone, OKA15. The clones are from the breeding collection of Agriculture and Agri-Food Canada and are maintained at the Benton Ridge Substation in Benton, New Brunswick and grown in the greenhouse at the Fredericton Research and Development Centre, Fredericton NB, Canada as described.^13^ The potato clones have distinctive tuber size, shape, and disease resistance. The parents of the potato clones are listed on Supplementary Table S1. While the pedigrees of the potato clones all include *S. tuberosum* they also include other *Solanum* species. The clone 12625-02 is derived from a cross of *Solanumandigena* x *S. tuberosum* and W5281.2 is from a cross of *Solanumphureja* x *S. tuberosum*. Additionally, breeder’s notes indicate that the pedigrees of 07506-01 and 11379-03 also have a mixture of *S. phureja* and *Solanumstenotomum*. It should be noted that in 2002 *S. andigena, S. phureja* and *S. stenotomum* were re-classified as cultivar groups within *S. tuberosum.*^14^ DW84-1457 has a background pedigree that includes wild species *Solanumacaule*, *Solanumdemissum*, *Solanumgourlayi* and *Solanumstoloniferum*. The OKA15 clone was propagated from a single seed from a US Genebank NRSP-6 *S. okadae* accession PI 458367.

### 2.2. DNA extraction and sequencing

Total genomic DNA was extracted from the leaves of each clone using the DNeasy Plant Mini Kit (Qiagen) following the manufacturer’s instructions for plant tissue. Sequencing of the ten clones was carried out using both 10X Genomics’ GemCode technology (https://www.10xgenomics.com/) and PacBio SMRT (Pacific Biosciences, Menlo Park, CA, USA) sequencing technology.

### 2.3. Mitogenome assembly and annotation

PacBio data was used to perform a *de novo* assembly of the 10 mitogenomes. First, the reads were aligned to a reference set created by concatenating the publicly available potato mitochondrial genome sequences [GenBank acc. MW122949-MW122985, MN104801-MN104803, MN114537-MN114539]^2^^,^^3^ using minimap2 v2.17.^15^ Mapped reads with a minimum alignment length of 2 kb were extracted and assembled using Canu v2.1.1.^16^ The genome size was set to 450 kb for the assembly with a minimum overlap and read length of 15 kb to resolve repeats. The contigs were manually assembled into scaffolds to obtain a complete mitogenome for each clone. Then the 10X reads were used for polishing the PacBio assemblies. The raw 10X reads were trimmed, and barcode error corrected using *Longranger basic.*^17^ The filtered reads were used to polish the final assemblies using pilon v1.23.^18^ The assembled mitogenome of each clone was annotated using GeSeq v2.02,^19^ with the GenBank accessions mentioned above as a reference. The annotations were manually curated to adjust gene boundaries using blast searches.^20^ Also, large repeat sequences were identified using self-blast searches.

### 2.4. Phylogeny

A phylogenetic tree was constructed from the coding sequences of the 33-common protein-coding genes (*atp1, atp4, atp6, atp8, atp9, ccmB, ccmC, ccmFc, ccmFn, cob, cox1, cox2, cox3, matR, mttB, nad3, nad4L, nad6, nad7, nad9, rpl10, rpl16, rpl2, rpl5, rps1, rps10, rps12, rps13, rps19, rps3, rps4, sdh3, sdh4*) for each clone. In addition, the same 33 genes from 13 published mitogenomes of a panel of diverse taxa,^2^^,^^21^ and two *S. tuberosum* cultivars (Cicero and Désirée)^3^ were included for this phylogenetic study. The coding sequences were extracted and concatenated from each mitogenome and used in constructing the phylogenetic tree. A multiple alignment was performed using MAFFT v7.475^22^ and then a maximum parsimony tree was inferred using paup v4.0a168 with 1000 bootstrap replicates.^23^ The tree was drawn using FigTree v1.4.4 (http://tree.bio.ed.ac.uk/software/figtree/). Furthermore, a plastome phylogeny was constructed for the same genomes except Cicero and Désirée (their plastome assemblies are not available) to determine congruency between both phylogenetic trees.^13^^,^^24^ The genome comparisons are visualized using MAUVE v20150226.^25^ The cytoplasm types were determined using *Insilco* PCR experiments.^26^

## 3. Results and discussion

### 3.1. Structure, organization, and gene content of potato mitochondrial DNA

**Figure 1:**
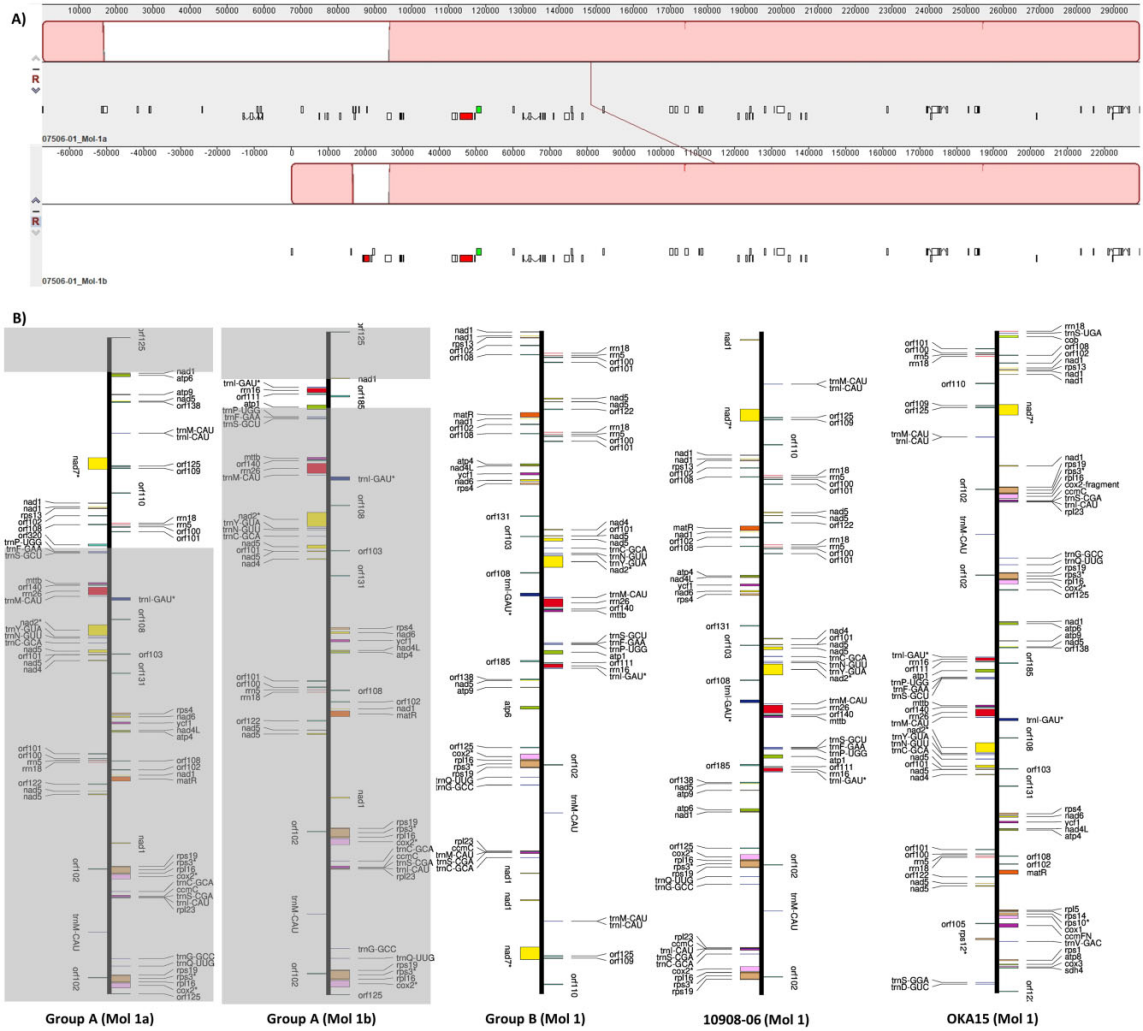
Comparison of molecule 1a and 1 b of group A mitogenomes (07506-01 is used to show the comparison). (A) Red blocks show the identical region between molecule 1a and 1 b, approximately 219 kb of sequence is identical between these two molecules. (B) Comparison of molecule 1 from each group, the grey boxes represent shared sequence between molecule 1a and 1 b of group A. The gene content and gene organization in molecule 1 of each group is represented here. The molecules are shown as linear maps for easier comparison.

We assembled the mitogenomes of nine diploid potato clones (mostly *S. tuberosum*) for which the plastomes were recently assembled^13^ using the pipeline described in Supplementary Fig. S1. In addition, we assembled the mitogenome of one clone of *S. okadae*. The results reveal a multichromosomal nature of their mitochondrial DNA, as each mitogenome was assembled into multiple individual molecules. The clones 07506-01, DW84-1457, 08675-21 and H412-1 were assembled into four circular molecules (Table 1). The mitogenome of these four clones is similar in sequence, structure, and organization and will therefore hereafter be referred to as group A. Molecules 1a and 1 b of group A share a common sequence of ∼219 kb, with ∼77.3 kb and ∼9.8 kb of unique sequence in molecule 1a and 1 b, respectively (Fig. 1). These two circular molecules are almost identical to a single linear mitogenome molecule (molecule 1) from two reference *S. tuberosum* clones that were also confirmed to have two alternate circular forms.^3^ It is possible that the molecule 1a and 1 b of group A may also exist as a single linear molecule *in vivo*, but there is no evidence of this in the current sequencing data. Furthermore, the mitogenomes of clones W5281-2, 12625-02, 12120-03 and 11379-03 were each assembled into three individual circular molecules with similar structure, and genome arrangements, and these four clones will be referred as group B. Finally, the mitogenomes of clone 10908-06 and OKA15 were also assembled into three independent circular molecules, but they were sufficiently different than those of group A and B. The molecular arrangement observed in OKA15 especially is very different from the rest of the mitogenomes (Table 1).

**Table 1:** Mitogenome size and conformations of 10 diploid potato clones (size in bp)

Genome	07506-01	H412-1	DW84-1457	08675-21	W5281.2	12625-02	12120-03	11379-03	10908-06	OKA15
	Group A				Group B					
Molecule 1	—	—	—	—	284,385	284,381	284,392	284,382	295,832	334,889
Molecule 1a	297,036	297,035	297,032	297,037	—	—	—	—	—	—
Molecule 1 b	229,589	229,586	229,598	229,608	—	—	—	—	—	—
Molecule 2	112,834	112,835	112,839	112,832	113,443	113,451	113,452	113,451	111,730	54,636
Molecule 3	49,239	49,238	49,238	49,242	49,167	49,169	49,170	49,182	49,265	49,277

The mitogenome of 075006-01, H412-1, DW84-1457 and 08675-21 have similar structure and organization with four circular molecules in each of them. Similarly, W5281.2, 12625-02, 12120-03 and 11379-03 clones have similar mitogenome structure with three individual circular molecules. Whereas the molecule 2 and molecule 3 of 10908-06 is similar to group A and group B molecule 2 and 3, with a different arrangement in its molecule 1. Finally, the OKA15 mitogenome is completely different from the rest of the mitogenomes. All the molecules listed are circular molecules.

**Figure 2:**
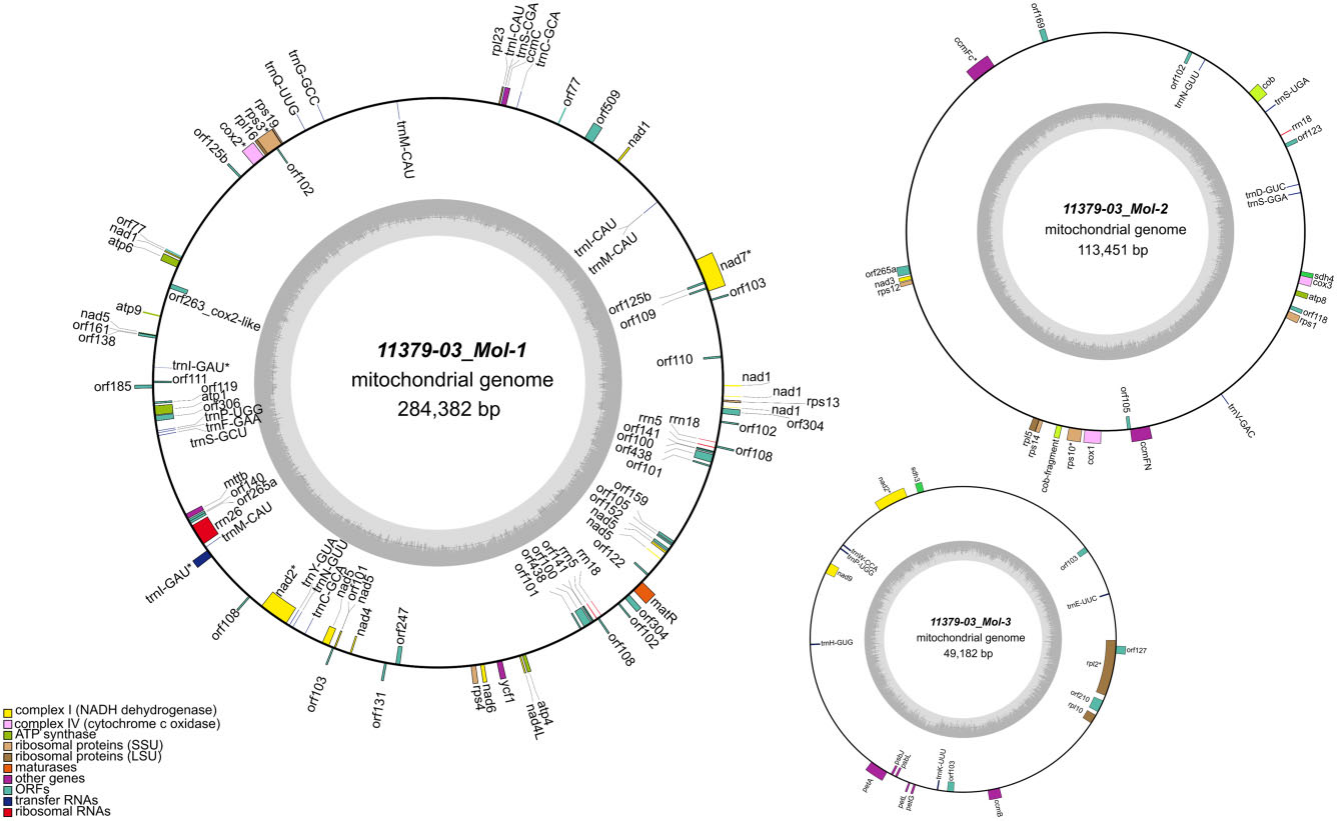
Arrangement and organization of genes in individual molecules of potato clone 11379-03. Similar gene organization was observed in the remaining group B clones (W5281-2, 12625-02, 12120-03 and 11379-03), whereas the rest of the mitogenomes have a different arrangement (group A clones 07506-01, DW84-1457, 08675-21, H412-1; clone 10908-06; and OKA15). The inner circles represent GC content, and the genes outside the outer circle are transcribed clockwise while the genes inside the outer circle are transcribed anti-clockwise.

Annotation of each mitogenome revealed a conserved set of genes that are common in potato mtDNA.^2^ Each mitogenome contains five ATP synthases (*atp1, atp4, atp6, atp8*, *atp9*), nine NADH dehydrogenases (*nad1, nad2, nad3, nad4, nad4L, nad5, nad6, nad7, nad9*), 11 ribosomal proteins (*rps1, rps3, rps4, rps10, rps12, rps13, rps19, rpl2, rpl5, rpl10, rpl16*), four cytochrome C biogenesis group genes (*ccmB, ccmC, ccmFC, ccmFN*), three cytochrome c oxidases (*cox1, cox2, cox3*), two succinate dehydrogenases (*sdh3, sdh4*), one cytcochrome c reductase (*cob*), one maturase (*matR*), and a transport membrane protein (*mttB*). All 10 mitogenomes have a functional *cob* gene while a truncated *cob* gene copy is also present in all but the OKA15 mitogenome. Three types of rRNAs (5S, 18S and 26S rRNA), and 22 tRNAs (corresponding to 16 amino acids) are present in each mitogenome (Fig. 2). Integration of plastid DNA into the mitogenome is not uncommon,^27^ and sequences of plastidial origin were found in the mitogenome of various land plants.^3^^,^^28^ We have also found multiple fragments of plastid sequences in each mitogenome, and some of these fragments contained *petA, petG, petL, psbJ, psbL* and *rpl23* genes of plastidial origin.^13^

In addition to the annotated genes, 50–54 open reading frames that are larger than 100 codons were annotated and compared between the mitogenomes. A chimeric hypothetical protein, *orf320* which carries a 5′ of *atp1* is only present in group A. This chimeric ORF is previously reported in potato,^3^ as well as in other species.^1^ A CMS-associated hypothetical protein, *orf137*, is present only in group A, and in the 10908-06 mitogenome. The *orf137* was previously found to be expressed in two potato mitogenomes,^3^ and its sequence is similar to the CMS-associated *orf456* of chili pepper.^5^ Furthermore, the *orf125*, is present only in group A clones, whereas *orf123* is absent only in 10908-06. It is also interesting to note that the *orf111* has internal stop codons in all the mitogenomes except in those of OKA15 and 10908-96, where there are no internal stop codons. Similar results regarding the *orf123* and *orf111* were previously found in other mitogenomes in a range of potato taxa as well.^2^

### 3.2. Repeat structure and genomic recombination in each mitochondrial DNA

Repeats are an integral part of plant mitogenomes and they range from several hundred bp to several thousand bp.^29^ Characterizing the repeat sequences is crucial in understanding the DNA maintenance mechanisms, evolution and recombination events in mitogenomes. Large repeats in plant mitochondria recombine very frequently and repeats of this nature (larger than 1000 bp) were identified in all the 10 mitogenomes (Table 2). A similar repeat structure was observed in the group A clones, as well as in 10908-06. Similarly, all the mitogenomes of the group B clones share a similar repeat structure. Interestingly, the repeat structure of the OKA15 mitogenome (from the wild species *S. okadae*) is very different compared with the rest of the mitogenomes (from *S. tuberosum* clones) (Table 2). We have named four different types of repeats in these mitogenomes as repeat R1–R4. These repeats were also reported previously, however a ∼10 kb repeat sequence previously found in three potato individuals (two accessions of *S. tuberosum* subsp. *andigena*, and *S. chaucha*) is not observed in any of these mitogenomes.^2^ Repeat R1 is the only repeat present in all clones, and in the group A mitogenomes the R1 repeats are inverted, whereas in OKA15 they are expanded. The presence of the R2 repeat in the mitogenomes of group A, 10908-06, and OKA15 leads to the inclusion of additional copies of *cox2, rpl16, rps19, rps3* and *orf102*, though in OKA15 the R2 repeat is reduced, resulting in a truncated copy of the second *cox2* gene.

**Table 2:** Repeat structure in each mitogenome (size in bp)

Genome	07506-01	H412-1	DW84-1457	08675-21	W5281.2	12625-02	12120-03	11379-03	10908-06	OKA15
R1a	11,916^a^ (1a, 1 b)	11,916^a^ (1a, 1 b)	11,916^a^ (1a, 1 b)	11,916^a^ (1a, 1 b)	11,916 (1)	11,916 (1)	11,917 (1)	11,917 (1)	11,916 (1)	13, 435 (1)
R1b	11,916^a^ (1a)	11,916^a^ (1a)	11,916^a^ (1a)	11,916^a^ (1a)	11,916 (1)	11,916 (1)	11,917 (1)	11,917 (1)	11,916 (1)	13, 435 (1)
R2a	7,502 (1a, 1 b)	7,502 (1a, 1 b)	7,502 (1a, 1 b)	7,502 (1a, 1 b)	—	—	—	—	7,502 (1)	7,076 (1)
R2b	7,502 (1a, 1 b)	7,502 (1a, 1 b)	7,502 (1a, 1 b)	7,502 (1a, 1 b)	—	—	—	—	7,502 (1)	7,076 (1)
R3a	—	—	—	—	4,513 (1)	4,513 (1)	4,513 (1)	4,513 (1)	—	—
R3b	—	—	—	—	4,513 (1)	4,513 (1)	4,513 (1)	4,513 (1)	—	—
R4a	1,589 (2)	1,589 (2)	1,589 (2)	1,589 (2)	1,589 (2)	1,589 (2)	1,589 (2)	1,589 (2)	1,589 (2)	—
R4b	1,589 (2)	1,589 (2)	1,589 (2)	1,589 (2)	1,589 (2)	1,589 (2)	1,589 (2)	1,589 (2)	1,589 (2)	—

Four repeats that are larger than 1000 bp were found in these mitogenomes. A similar repeat structure was found in group A clones, and 10908-06. All the group B clones have the same repeat structure. OKA15 repeat structure is very different from the rest of mitogenomes.

a Represents inverted repeat sequences. Which repeat is present in which molecule of that particular genome is written inside the parentheses. Repeats R1, and R2 or R3 are present in molecule 1 of each mitogenome. R2 repeat in group A is present in both molecule 1a and 1 b, whereas a second copy of R1 repeat sequence (R1b) is missing in molecule 1 b. R4 repeat is present in molecule 2 of each mitogenome, except in OKA15 where it lacks R4 repeat.

**Figure 3:**
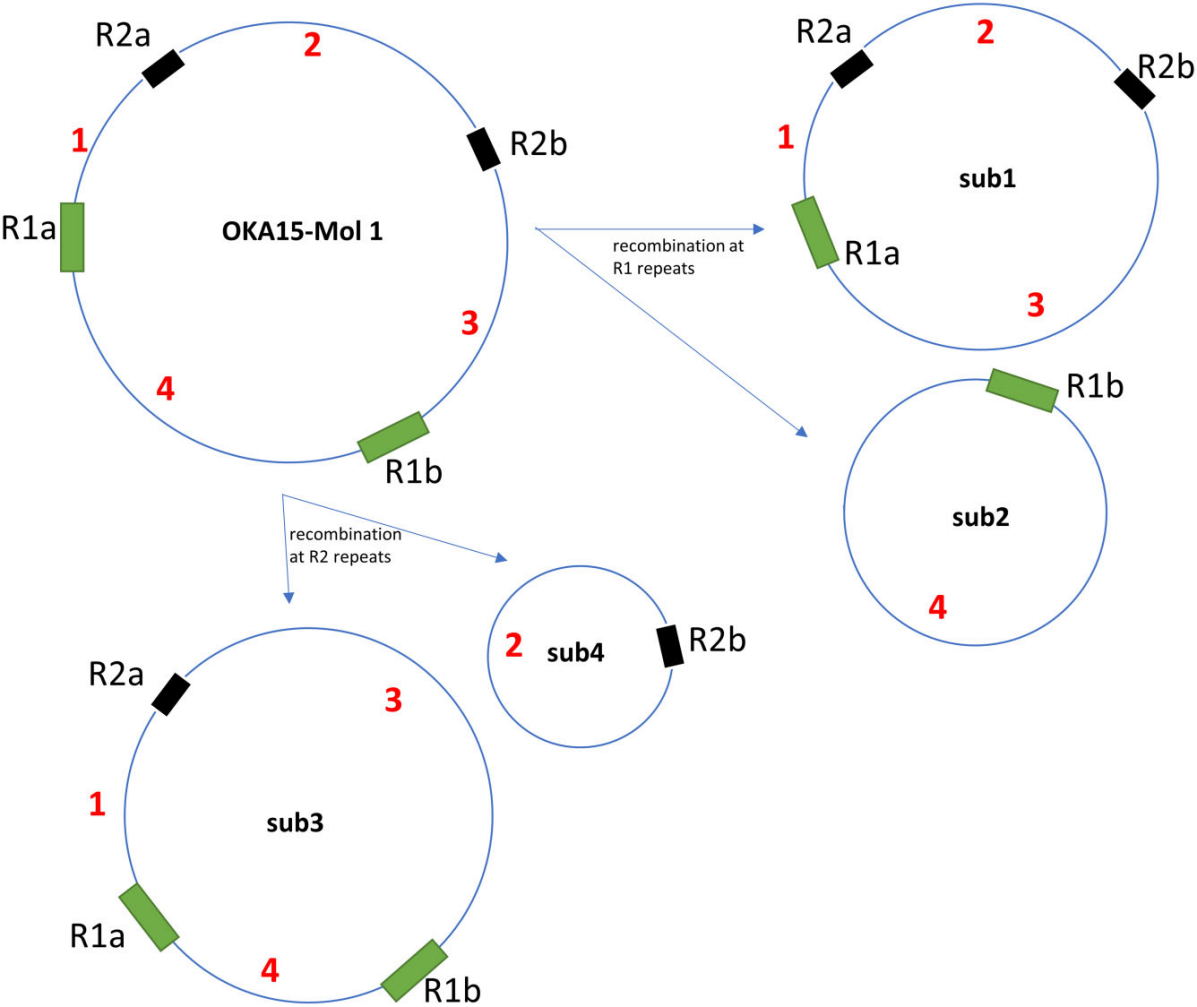
Representation of recombination events in OKA15 mitogenome molecule 1, and its sub-genomic circles confirmed by reverse read mapping. Similar recombination patterns were observed in molecule 1 and molecule 2 of each mitogenome. All the observed recombination events are derived from the R1, R2, R3 and R4 repeat sequences.

Recombination in plant mitochondria is very frequent because of the high repeat content, resulting in multiple isoforms, loss of synteny and genomic rearrangements.^11^^,^^29^ The large repeat sequences are mainly involved in recurrent, and reversible recombination events. One of the many advantages of long-read sequencing is the ability to detect recombination events in mitogenomes.^1^ Here, we were able to identify patterns of recombination in each mitogenome with the help of PacBio data (Supplementary information). These recombination events were detected in individual molecules only, and no evidence was observed to support the ‘master circle’ view of these mitogenomes. The molecule 1 of each mitogenome has sub-genomic circles formed by the repeats R1, and either R2 or R3 (Fig. 3, Supplementary Figs S2–S7). Similarly, molecule 2 of each mitogenome, except OKA15, also subjected to recombination at the R4 repeat sequence (Supplementary Figs S4–S7). Figure 3 represents the recombination in OKA15 molecule 1 and its multiple isoforms. A reverse read mapping approach was used to confirm the recombination events in each mitogenome (Supplementary information). The presence of four sub-genomic circles was confirmed for group B, 10908-06, and OKA15 molecule 1, while the molecule 1a of group A mitogenomes has two sub-genomic circles formed by R2 repeat sequence, and alternate arrangements formed by R1 inverted repeats. Molecule 1 b (group A) has only two sub-genomic circles at R2 repeat, and it lacks R1 repeat sequence. In addition, molecule 2 of each mitogenome, except that of OKA15, has two sub-genomic circles with ∼45 kb and ∼67 kb sequence.

### 3.3. Mitogenome comparisons and phylogenetic analysis

**Figure 4:**
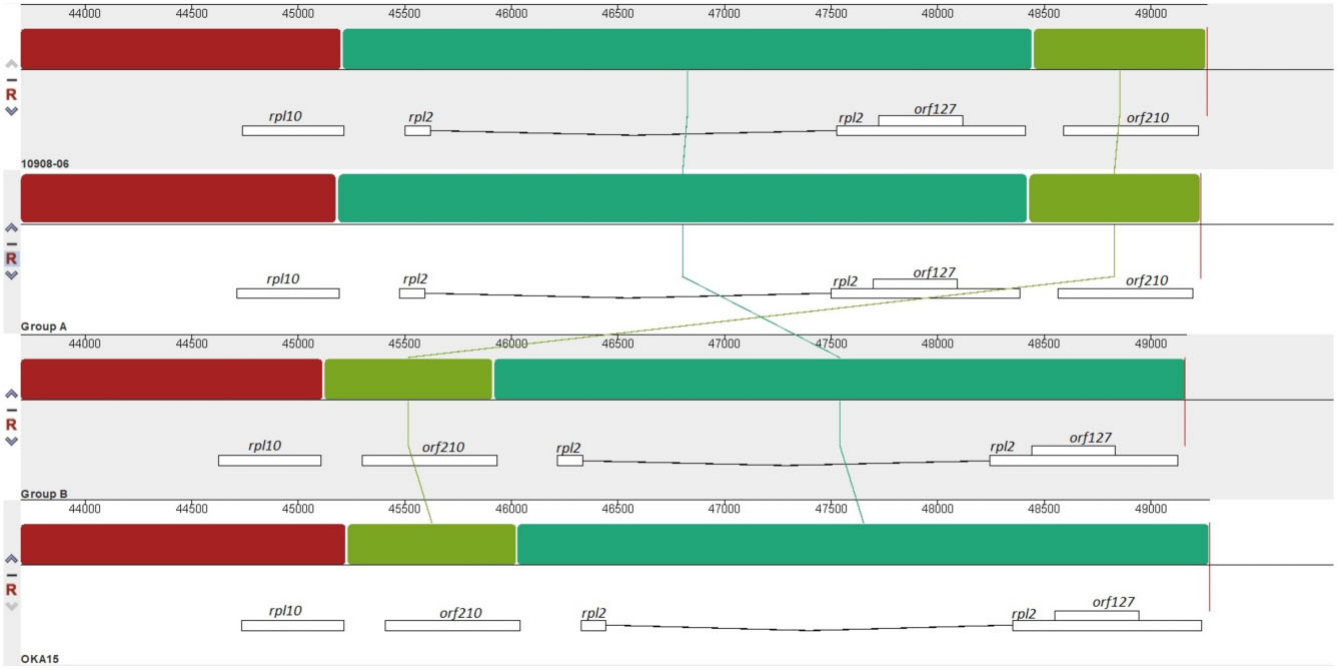
Comparison of mitogenome molecule 3 from potato clone group A, group B, 10908-06 and OKA15. An important difference between molecule 3 of each mitogenome is the translocation of light green block with ∼774 bp region having *orf210*.

The mitogenomes were compared to determine genomic rearrangements. A comparison between the molecule 1 of each mitogenome showed extensive rearrangements between them (Fig. 1b, Supplementary Fig. S8). Similarly, comparison between molecule 3 of each mitogenome revealed a translocation of ∼774 bp region in group B and OKA15 compared to group A and 10908-06 (Fig. 4). The translocated region is located between *rpl10* and *rpl2* genes in group B and OKA15 (Fig. 4), whereas the same region is located upstream of *rpl2* gene in group A and 10908-06. Annotations revealed the presence of a hypothetical protein named *orf210* in the translocated region. Interestingly, this region was previously used as a marker for determining mitochondrial DNA types in potato.^30^

**Figure 5:**
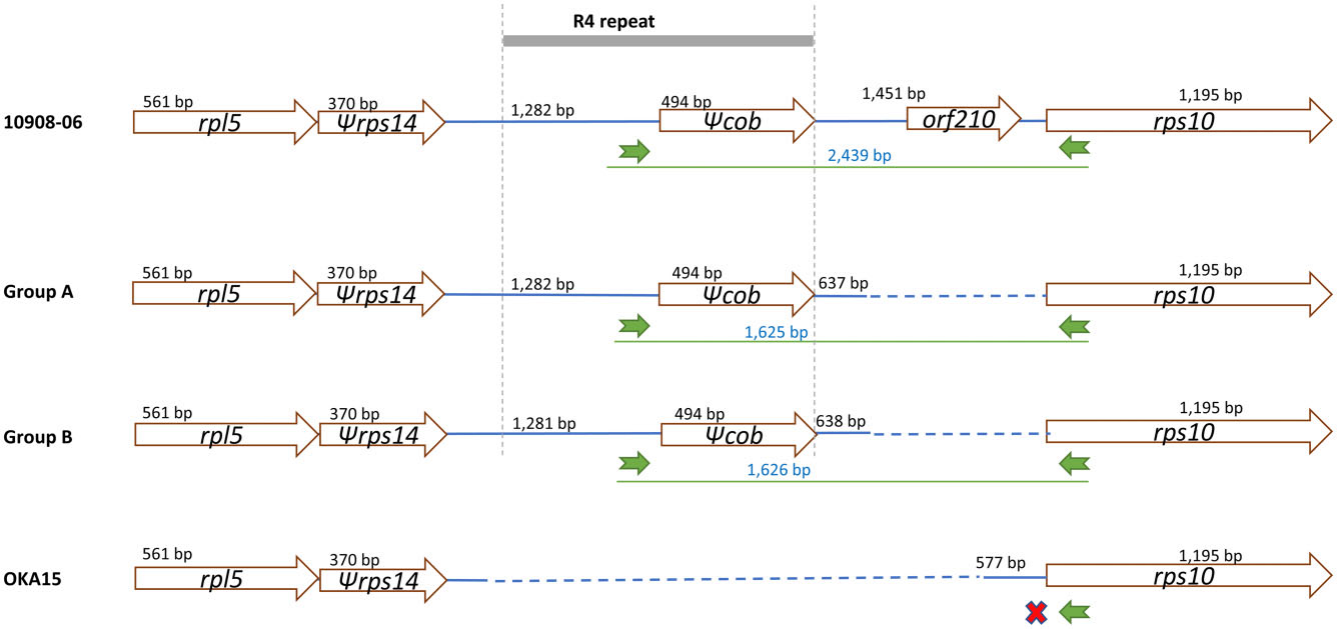
Arrangement of the region between *rps14* and *rps10* of potato clone group A, group B, 10908-06 and OKA15. A lot of variation is observed in this region, for example the lack of a *cob*-fragment in OKA15 due to a lack of the R4 repeat, and the presence of an additional *orf210* between the *cob*-fragment and *rps10* of 10908-06. The green arrows represent the location of PCR primers to detect mtDNA type, with their PCR product size. The red cross indicates no PCR product for OKA15.

Molecule 2 of each mitogenome is very similar in structure and gene organization, except in molecule 2 of OKA15. However, the molecule 2 from these nine mitogenomes is mapped to both molecule 1 and molecule 2 of OKA15 (Supplementary Fig. S9). An interesting region of observation is the arrangement of the *Ψrps14*-*Ψcob*-*rps10* genes. While the mitogenome of OKA15 lacks *Ψcob*, the others have differences in the intergenic region between *Ψcob* and *rps10* (Fig. 5). In 10908-06, the region between *Ψcob* and *rps10* has a copy of the above-mentioned translocated region that contains *orf210* making it ∼814 bp longer compared to group A and B, whereas group A and group B have an intergenic region of 637 and 638 bp, respectively. These arrangements were observed in previous studies as well and used as markers for identifying potato mtDNA types.^2^^,^^3^^,^^9^^,^^31^^,^^32^ Nevertheless, the presence of *orf210* in the *Ψcob-rps10* region was not mentioned in the previous studies.

**Figure 6:**
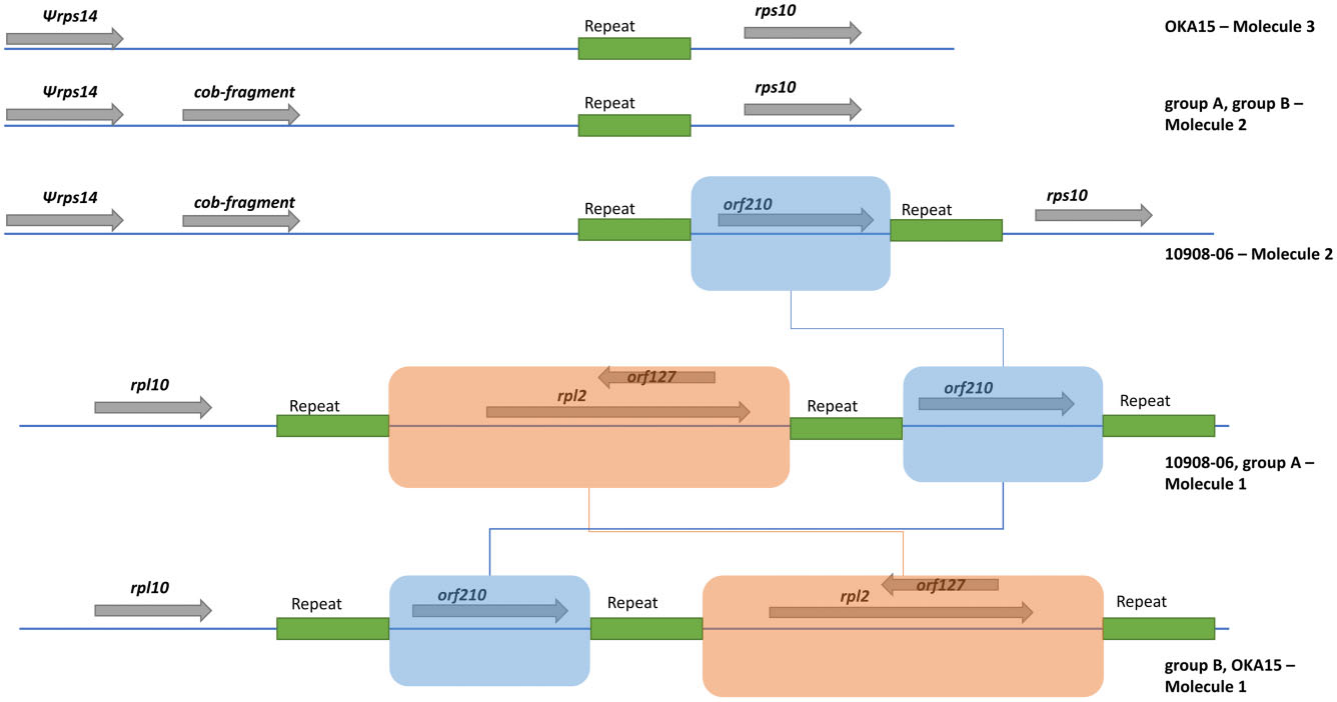
Schematic representation of a translocation of a ∼774 bp region in potato mitogenome molecule 3, and insertion of the same region in molecule 2 of potato clone 10908-06. The schematic shows the presence of a short repeat sequence (green block) of 40 bp in the translocated region in molecule 3 of each mitogenome as well as in the insertion region of 10908-06 molecule 2. This repeat sequence is present on both sides of the translocated region (blue block). Presence of this repeat sequence suggests a potential recombination that may have occurred between molecules.

A short repeat sequence of 40 bp was found on both sides of the translocated region in each molecule 3 (Fig. 6). Interestingly, the same repeat sequence was found in the molecule 2 of 10908-06 which explains the insertion of the translocated region in 10908-06 molecule 2 (Fig. 6). It is possible that a recombination event might have occurred at this repeat sequence that resulted in the translocation, and insertion of sequences.

The green arrows in Fig. 5 indicate the PCR primers that were designed previously to detect mtDNA types in potato.^30^ Using computational PCR methods, while no PCR product was observed for OKA15, PCR products sized ∼2.4 kb for 10908-06, and ∼1.6 kb for group A and group B were observed. These correspond to the γ-type mtDNA (absence for OKA15), α-type mtDNA (2.4 kb for 10908-06) and β-type mtDNA (1.6 kb for group A and B), respectively, according to.^32^ Taking these results together with the previous study of chloroplast (cpDNA) types of these potato clones,^13^ the group A clones have a T/β cytoplasm, group B clones have a S/β cytoplasm, 10908-06 have a W/α cytoplasm, and OKA15 have a W/γ cytoplasm. The cytoplasm types found in our study were also found previously in many of the potato populations,^7^ except S/β cytoplasm. Previous studies have shown that W/γ cytoplasm is strongly correlated with tuber starch content, resistance to late blight, and male sterility.^7^^,^^32^

**Figure 7.**
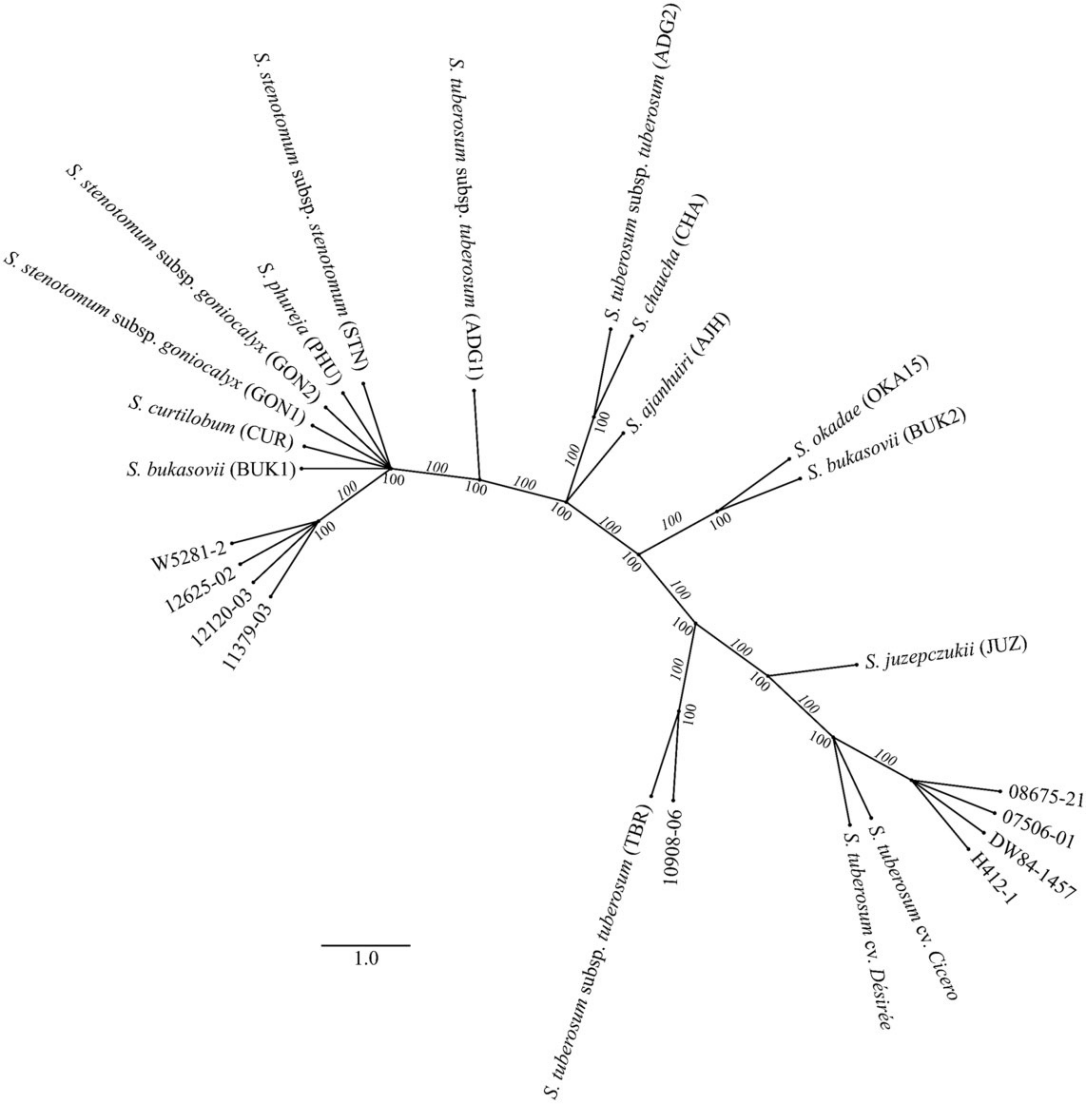
A phylogenetic reconstruction based on the mitogenomes from 10 diploid potato clones in this study, and from 15 previously published potato taxa. All the group B clones are in a single clade along with *S. phureja* (PHU), *S. stenotomum* subsp. *stenotomum* (STN), *S. stenotomum* subsp. *goniocalyx* (GON1), *S. stenotomum* subsp. *goniocalyx* (GON2), *S. curtilobum* (CUR) and *S. bukasovii* (BUK1). Similarly, all the group A clones are grouped together along with *S. tuberosum* cv. Cicero and Désirée. The wild species OKA15 is grouped with *S. bukasovii* (BUK2), whereas 10908-06 is grouped with *S. tuberosum* subsp. *tuberosum* (TBR).

A phylogenetic tree was constructed for these 10 mitogenomes with an additional 13 previously published mitogenomes of diverse potato taxa,^2^ and two *S. tuberosum* cultivars.^3^ The clustering of group A mitogenomes is observed in a single clade (Fig. 7). Interestingly, clustering of the two *S. tuberosum* cultivars Cicero and Désirée closer to group A clones corelates with the sequence similarity found between them. Similarly, group B mitogenomes are also clustered together in one clade along with *S. phureja* (PHU), *S. stenotomum* subsp. *stenotomum* (STN), *S. stenotomum* subsp. *goniocalyx* (GON1), *S. stenotomum* subsp. *goniocalyx* (GON2), *Solanumcurtilobum* (CUR), and *Solanumbukasovii* (BUK1). There is a close relationship between 10908-06 and *S. tuberosum* subsp. *tuberosum* (TBR), as well as between OKA15 and *S. bukasovii* (BUK2). A plastome phylogenetic tree was built for the same species to observe the similarities/differences that could account for potential paternal mitochondrial DNA leakage. However, all the mitogenomes have similar groupings in both phylogenetic trees, except for *Solanumjuzepczukii* (JUZ), which is grouped with *Solanumajanhuiri* (AJH) in the plastome phylogeny but is closer to group A genomes in the mitogenome phylogeny (Supplementary Fig. S10). There were three clones where the paternal clone was also included in the study—12625-02, 08675-21 and 10908-06 (Supplementary Table S1). While 10908-06 clustered away from its paternal parent clone (H412-1), 12625-02 and 08675-21 did cluster together with their paternal parent clones, 11379-03 and 07506-01, respectively. Cytoplasms are inherited maternally, and similarity of paternal mitogenomes with progeny clones suggests potential leakage of paternal mitogenomes.

### 3.4. Conclusion

In this study, the mitogenomes of 10 diploid potato clones were assembled, annotated, and compared to explore their genetic diversity. Multichromosomal type arrangement in these mitogenomes were observed, consistent with the results from previous studies on potato mitogenomes.^2^^,^^3^ Novel structures and sequence rearrangements that were not reported previously were observed in some of these mitogenomes. Hence, it is necessary to sequence and analyse more mitogenomes to identify novel genomic structures to determine evolutionary patterns. Apart from the differences in gene copy number and presence/absence of certain unique ORFs, each mitogenome consists a set of conserved genes that are common in potato. Future studies focusing on the functional analysis of the novel ORFs could help in understanding the nuclear–cytoplasmic interactions that affect CMS in potato. Overall, four repeats (>1000 bp) were found in these mitogenomes with differences in their repeat structure. Interestingly, the repeat structure in the wild *S. okadae* is completely different from the rest of the mitogenomes in this study and those in previously reported studies.^2^ Thanks to long reads, recombination patterns were also detected in each mitogenome, proving the importance of long reads to successfully identify multiple isoforms in mitogenomes. Finally, the phylogenetic analyses suggested a potential paternal leakage of mitogenome in 12625-02 and 08675-21, however further studies are required to confirm this.

## Supplementary Material

dsab009_Supplementary_Data
