# Supplementary material for: The complete mitogenome assemblies of 10 diploid potato clones reveal recombination and overlapping variants
Source: DNA Res. 2021 Jul 10;28(4):dsab009. doi: 10.1093/dnares/dsab009 (PMC8386665; doi:10.1093/dnares/dsab009)
Supplement: dsab009_Supplementary_Data [file dsab009_supplementary_data.pdf]

Supplementary information

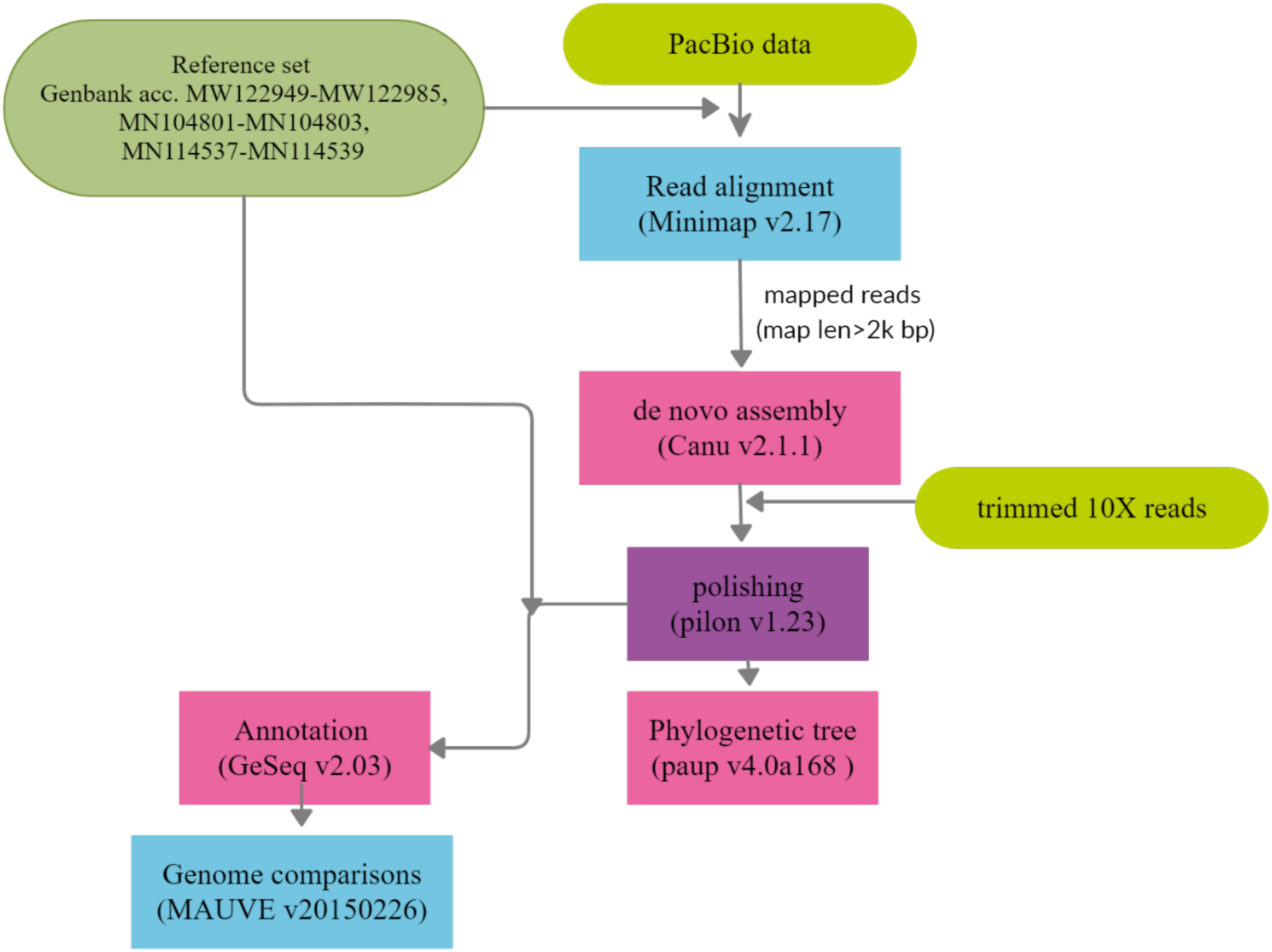

Supplemental Figure 1: Schematic representation of the pipeline used in this study to assemble and analyse mitogenomes of ten potato clones.

Recombination detection

We have used reverse read mapping to identify inter-molecular and intra-molecular recombination events in each mitogenome. The contigs obtained for each mitogenome suggested recombination patterns at repeat sequences. Further analysis of read mappings at these repeats provided additional evidence for recombination. For example, OKA15 mitogenome was assembled into three independent circular molecules with two large repeats (R1, and R2) in molecule 1. Four possible sub-genomic circles can be generated by recombination at

these two repeats in OKA15 molecule 1. We counted the number of reads spanning each repeat sequence plus 1000 bp sequence on each side of the repeat. First, we have confirmed the arrangement of molecule 1 by mapping the corrected reads from canu (Supplemental Figure 2). The boxes indicate the repeat sequences, and the arrows represent the sequence spanning the repeats. The number on top of the arrows indicate the read count supporting the arrangement.

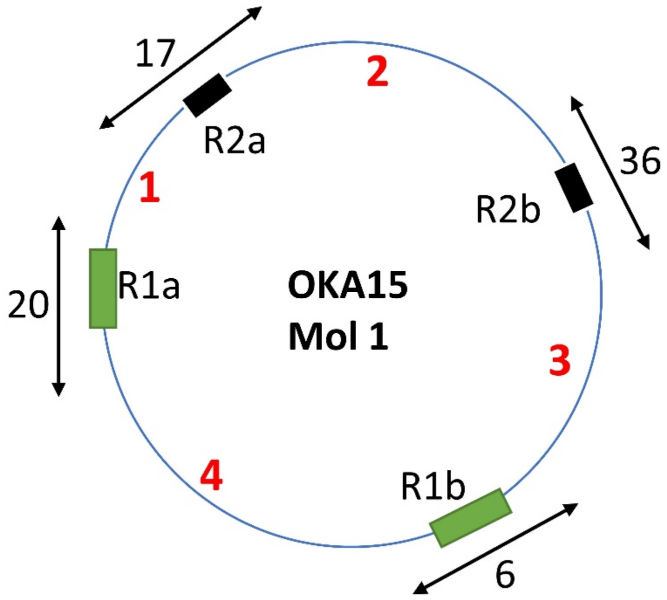

Supplemental Figure 2: Arrangement and repeats in molecule 1 of OKA15, with number of reads spanning each repeat sequence confirming the arrangement of the molecule 1.

Next, we have confirmed four possible sub-genomic circles generated by recombination at R1, and R2 repeats. Recombination at R1 repeat will give rise to sub genomes 1 & 2. They both have 12 and 19 corrected reads supporting the recombination. Similarly, sub genomes 3 & 4 were confirmed by read count at R2 repeat sequence (Supplemental Figure 3).

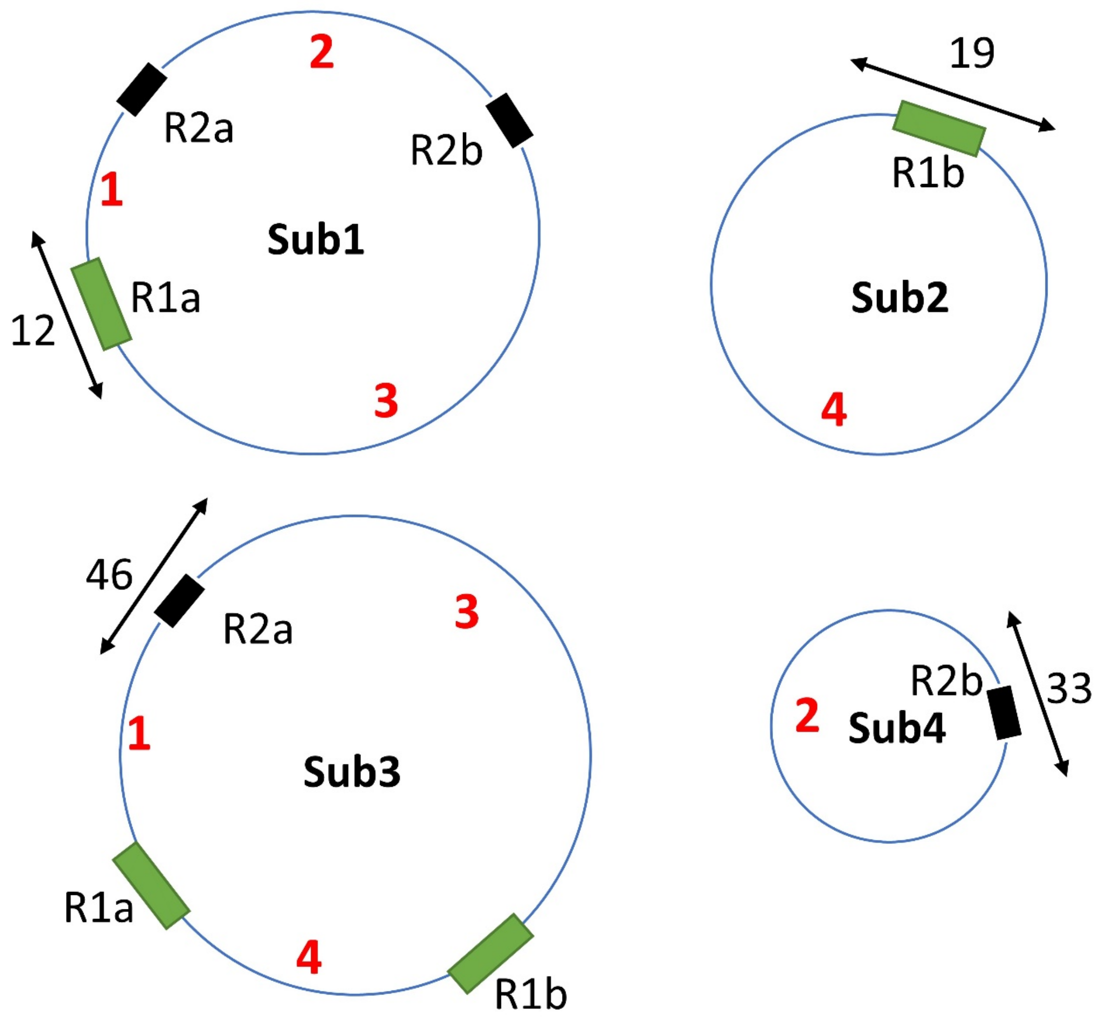

Supplemental Figure 3: Arrangement of sub-genomic circles in molecule 1 of OKA15, with number of reads spanning each repeat sequence and confirming the presence of sub-genomic circles. The sub-genomic circles 1 and 2 are formed by recombination at R1 repeat sequence, whereas sub-genomic circles 3 and 4 are formed by recombination at R2 repeat sequence.

Similar recombination patterns were observed for molecule 1 and molecule 2 in each mitogenome. Recombination events observed at R1, and R2 repeats for group A, 10908-06, and OKA15, whereas at R1, and R3 for group B mitogenomes. Also, recombination at R4 repeat sequence in molecule 2 of each mitogenome, except OKA15, leading to two sub genomic circles of length ~45 kbp and ~67 kbp (Supplemental Figure 4).

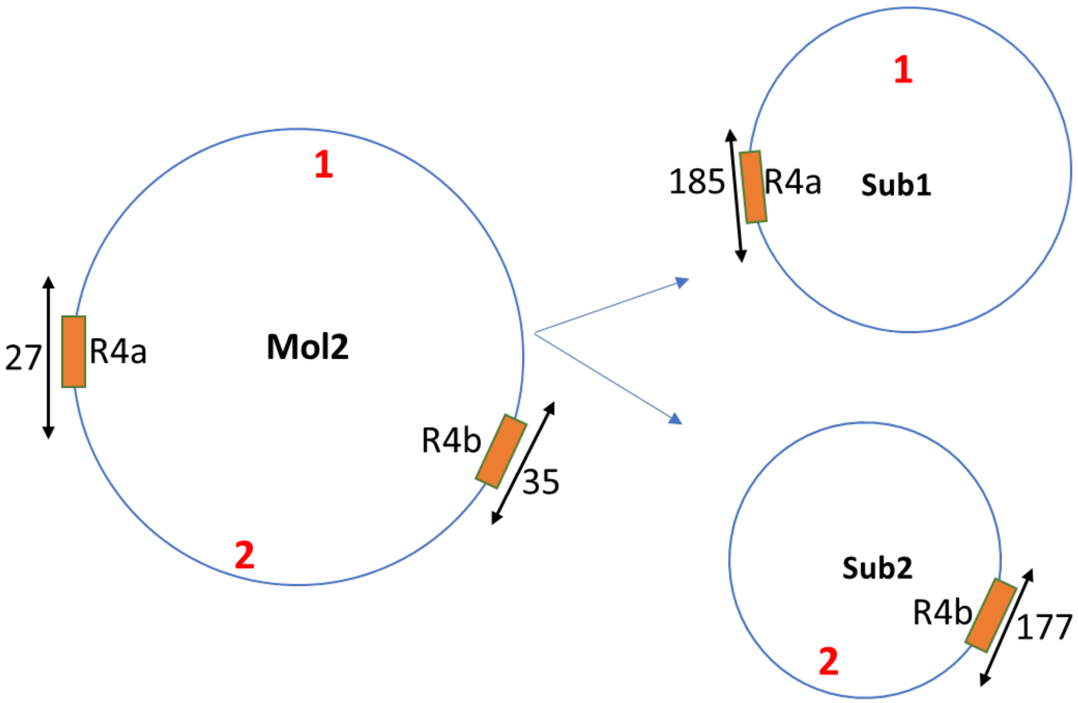

Supplemental Figure 4: Arrangement of sub-genomic circles in molecule 2 of 07506-01, with number of reads spanning each repeat sequence and confirming the presence of sub-genomic circles. The sub-genomic circles are formed by recombination at R4 repeat sequence.

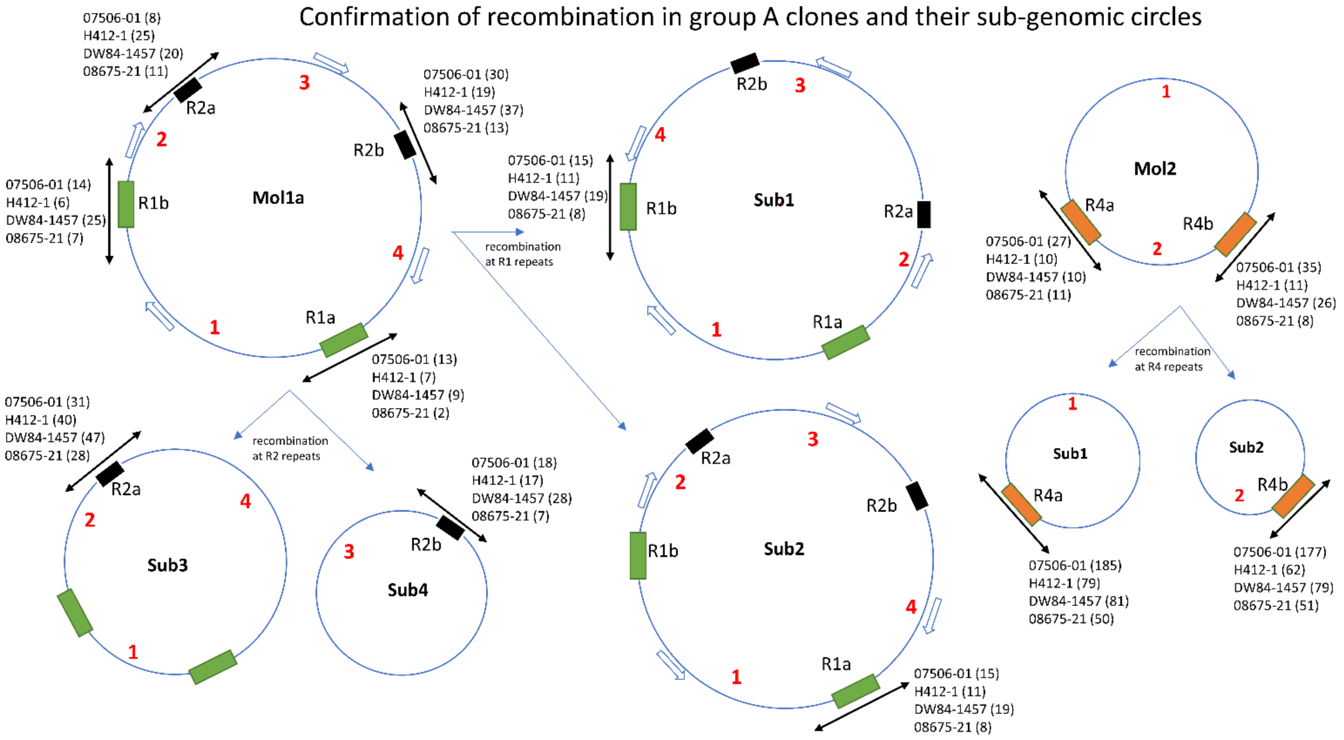

Supplemental Figure 5: All the recombination events at the repeat sequences R1, R2, and R4 in group A mitogenomes are confirmed by read mappings. The figure shows an arrangement of sub-genomic circles in group A mitogenomes with number of reads spanning each repeat sequence and confirming the presence of sub-genomic circles. The number of reads spanning at each repeat for each mitogenome is given in brackets.

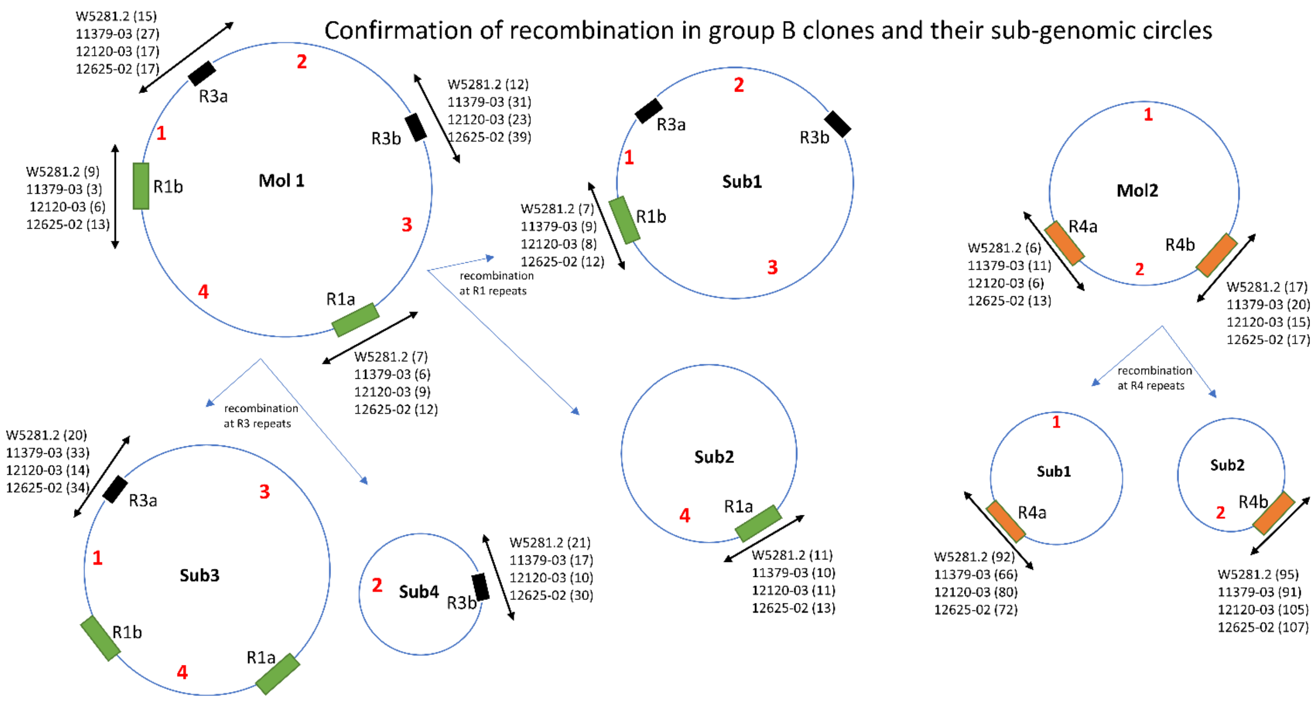

Supplemental Figure 6: All the recombination events at the repeat sequences R1, R3, and R4 in group B mitogenomes are confirmed by read mappings. The figure shows an arrangement of sub-genomic circles in group B mitogenomes with number of reads spanning each repeat sequence and confirming the presence of sub-genomic circles. The number of reads spanning at each repeat for each mitogenome is given in brackets.

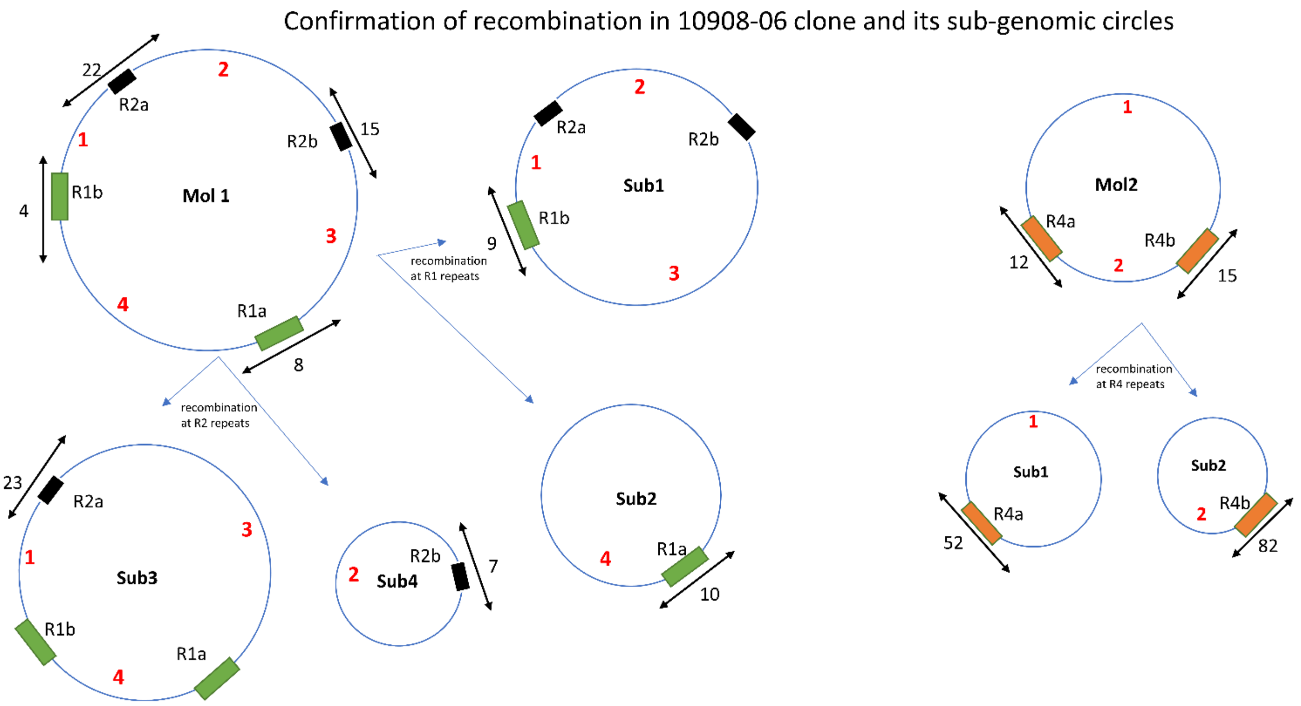

Supplemental Figure 7: All the recombination events at the repeat sequences R1, R2, and R4 in 10908-06 mitogenome are confirmed by read mappings. The figure shows an arrangement of sub-genomic circles in 10908-06 mitogenome with number of reads spanning each repeat sequence and confirming the presence of sub-genomic circles.

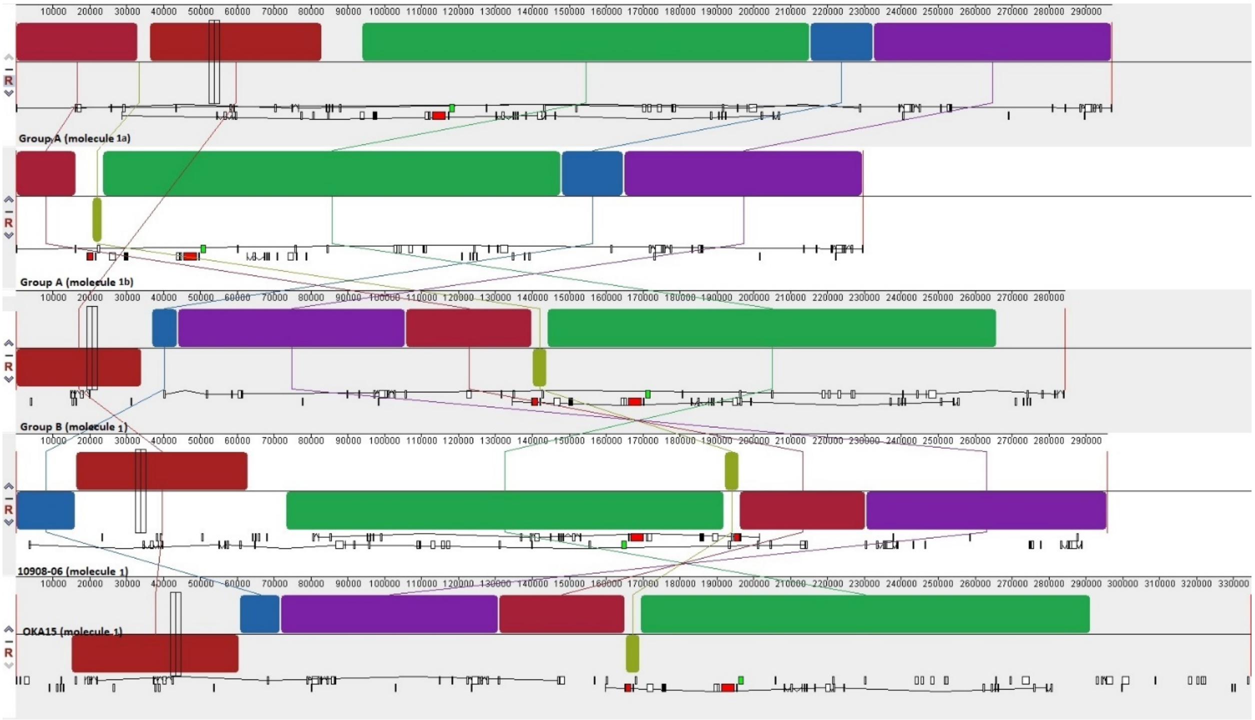

Supplemental Figure 8: Comparison of molecule 1 of each mitogenome, a lot of rearrangements are observed between these molecules.

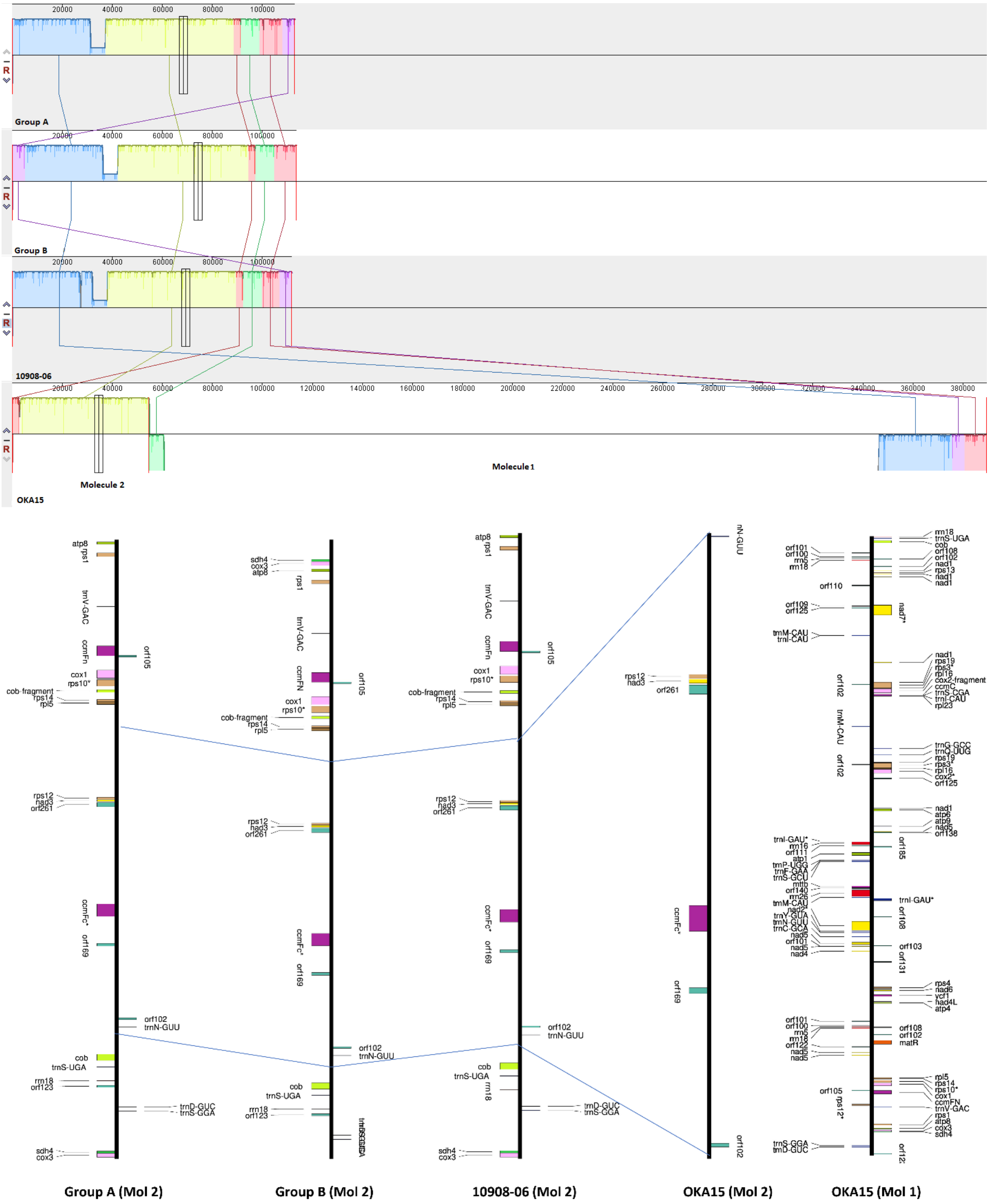

Supplemental Figure 9: Comparison of molecule 2 of each mitogenome, similar arrangement is seen in group A, group B, and 10908-06. Whereas this molecule 2 is distributed over molecule 1 and molecule 2 of OKA15.

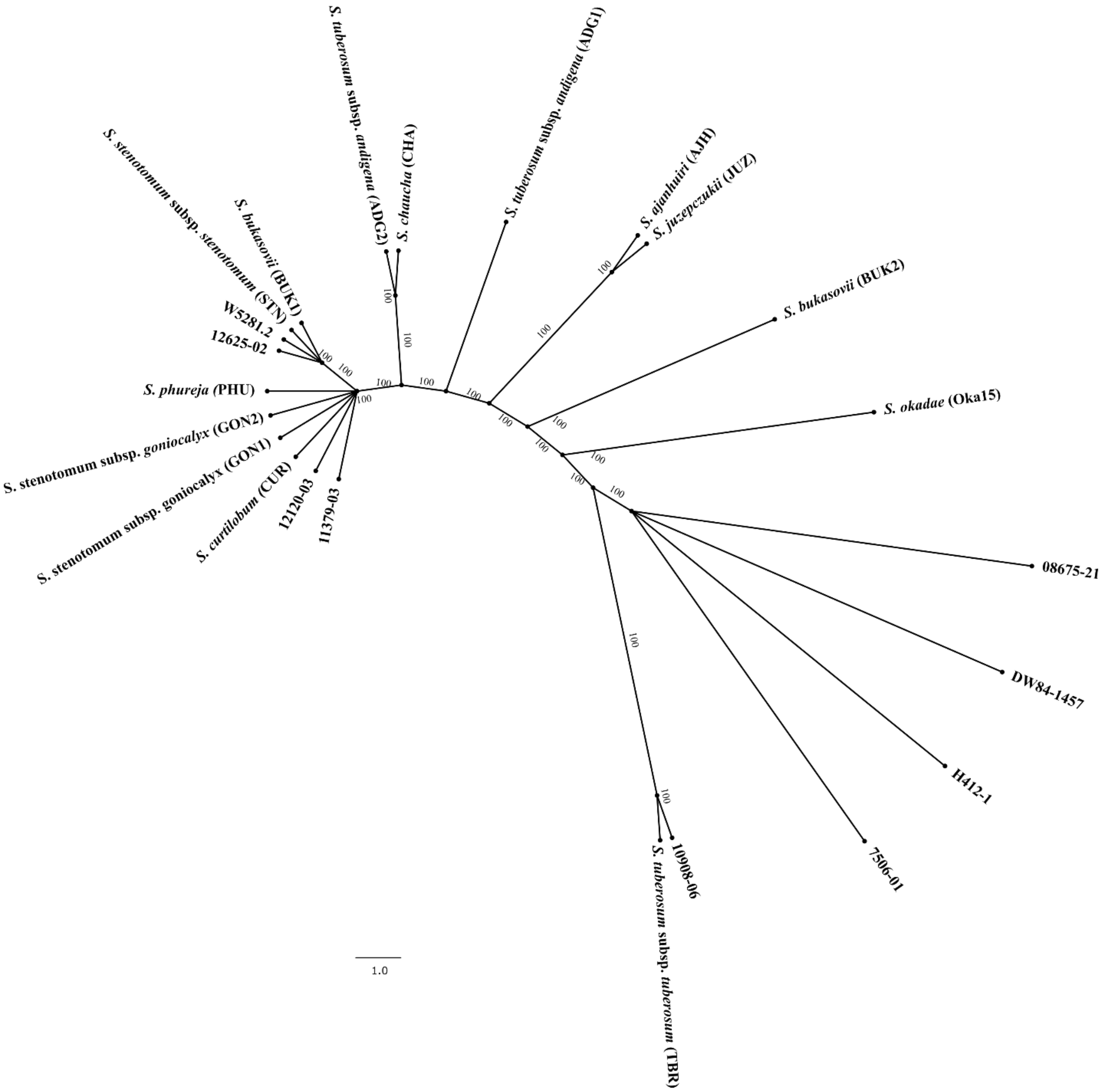

Supplemental Figure 10: A phylogenetic tree constructed from the plastome of the same individuals used for the mitogenome phylogeny. Similar groupings are observed in the plastome phylogeny as well, except for JUZ.

Supplemental Table 1: Parents of potato diploid clones.

Clone number	Parents	cpDNA type	mtDNA type
**H412-1**	06026-08 x (bulk diploid S. tuberosum)	T	β
**10908-06**	09507-03 x H412-1	W	α
**07506-01**	W9306.2 x (bulk diploid hybrids)	T	β
**08675-21**	06824-02 x 07506-01	T	β
**11379-03**	BPH32-05 x 09941-05	S	β
**12625-02**	2x(V-2)7 (S. andigena) x 11379-03	S	β
**W5281.2**	phu 195198 (S. phureja) x W1	S	β
**12120-03**	09113-11 x 09753-01	S	β
**DW84-1457**	DW81-1470 x DW80-2031	T	β
**OKA15**	wild species	W	γ
